# Storing, linking, and mining microarray databases using SRS

**DOI:** 10.1186/1471-2105-6-192

**Published:** 2005-07-27

**Authors:** Antoine Veldhoven, Don de Lange, Marcel Smid, Victor de Jager, Jan A Kors, Guido Jenster

**Affiliations:** 1 Department of Urology, Josephine Nefkens Institute, Erasmus MC, P.O. Box 1738, 3000 DR Rotterdam, The Netherlands; 2 Medical Oncology, Erasmus MC, Rotterdam, The Netherlands; 3 Bioinformatics, Erasmus MC, Rotterdam, The Netherlands; 4 Medical Informatics, Erasmus MC, Rotterdam, The Netherlands

## Abstract

**Background:**

SRS (Sequence Retrieval System) has proven to be a valuable platform for storing, linking, and querying biological databases. Due to the availability of a broad range of different scientific databases in SRS, it has become a useful platform to incorporate and mine microarray data to facilitate the analyses of biological questions and non-hypothesis driven quests. Here we report various solutions and tools for integrating and mining annotated expression data in SRS.

**Results:**

We devised an Auto-Upload Tool by which microarray data can be automatically imported into SRS. The dataset can be linked to other databases and user access can be set. The linkage comprehensiveness of microarray platforms to other platforms and biological databases was examined in a network of scientific databases. The stored microarray data can also be made accessible to external programs for further processing. For example, we built an interface to a program called Venn Mapper, which collects its microarray data from SRS, processes the data by creating Venn diagrams, and saves the data for interpretation.

**Conclusion:**

SRS is a useful database system to store, link and query various scientific datasets, including microarray data. The user-friendly Auto-Upload Tool makes SRS accessible to biologists for linking and mining user-owned databases.

## Background

The extraction of information from data generated by high-throughput experiments in genomics and proteomics has been likened to "attempting to drink from a fire hose". We are flooded with information on many levels such as whole genome DNA sequences, RNA expression, protein-protein interactions, protein modifications, and more. All this information is accessible in very different formats, ranging from well-organized curated gene sequences to unstructured free text in scientific literature. A system that can manage, link and query these heterogeneous types of datasets is therefore extremely valuable. The Sequence Retrieval System (SRS) is such a unified database system in which numerous different scientific databases have already been integrated [1].

Of special interest are data from high-throughput RNA expression microarrays [2,3]. Many of these datasets are freely available and, like information stored in other scientific databases, are from different platforms [4,5]. Integrating and mining these databases strongly facilitates the analysis of genes of interest but will also support discovery of disease markers, drug-targets and new knowledge in general [6-9]. One such platform is Oncomine, which has integrated many different microarray datasets, focussing on human cancer [10]. Additionally, standardized microarray depositories such as GEO (Gene Expression Omnibus) [11], ArrayExpress [12], and CIBEX [13] do or will soon provide options to browse and query the datasets [14-17]. No doubt, other platforms will be developed focussing on the integration of microarray data. If started from scratch, these initiatives will likely be limited in their direct linkage to other heterogeneous biological databases due to the laborious task of making those connections and programming the single and batch-wise query options. The universality and the availability of numerous scientific databases that have already been integrated in SRS make it a useful platform for integrating microarray databases. Although the SRS interface to query databases is quite user friendly, other aspects of working with SRS are not. These include (i) uploading microarray datasets, (ii) database security including setting user access, (iii) linking databases, (iv) generating standard views, and (v) communication with other programs such as statistical and clustering software. The current SRS interface has a major disadvantage in that it is not designed to perform complex calculations on the fly. This means that any microarray dataset to be uploaded must have all ratio and statistical calculations performed upfront. For example, once in SRS, one cannot change ratios from log10 to log2 or add an extra field per gene by dividing expression data of all "normal" by all "cancer" samples. However, software programs that perform calculations, statistical evaluations, clustering, protein domain predictions, homology searches, and more, can communicate with SRS. Interfaces can be generated that retrieve data from SRS, perform the required action and if desired, store the results in SRS. Alternatively, SRS allows direct integration of programs such as the BLAST and FASTA homology searches and the SRS-EMBOSS (European Molecular Biology Open Software Suite) tools [18,19].

Generating a database in which heterogeneous datasets are integrated is a challenge in itself. However, retrieving statistically meaningful data by comparing datasets from different sources, platforms and designs is particularly difficult [20]. There is a fast growing body of publications on microarray cross-platform comparisons, mainly showing how this can be achieved in very many different ways [8,21-26]. Statistical evaluations of data within a dataset of sufficient technical and biological replicates, are better defined and can be implemented per dataset within a database system [27,28]. The strategies and applications we discuss here to link, store and query scientific datasets in SRS, do not go beyond processed individual datasets and do not include cross-platform dataset integrations. We assume that each uploaded dataset consists of high-quality data and has been processed correctly.

In this paper we describe strategies to incorporate microarray databases into SRS and provide a database upload tool. Using the program Venn Mapper as an example, we show the possibility to automatically retrieve the stored microarray data from SRS for external statistical evaluation.

## Implementation

### Auto-Upload Tool

**Figure 1:**
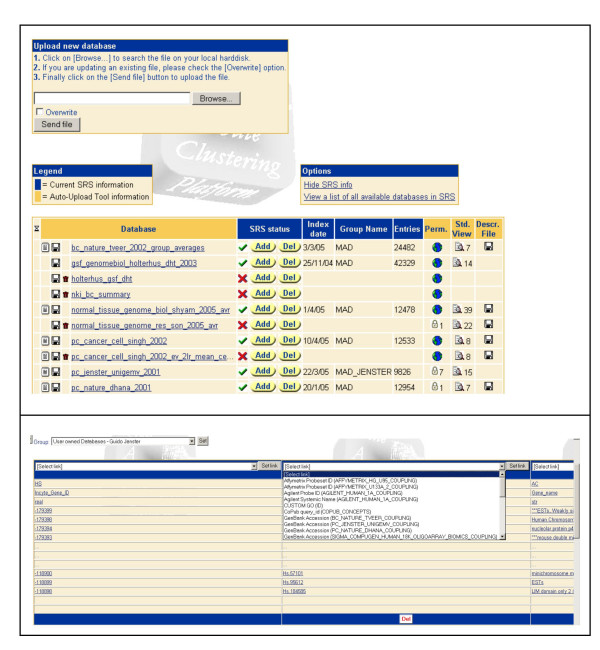
Screenshots of the database Auto-Upload Tool for SRS. Within the Auto-Upload Tool, a user can import a database and define links, SRS subdirectory, user access, a dataset description, and an SRS standard view.

In order to import microarray databases into SRS (version 7.1.3), an Auto-Upload Tool was built (Figure 1). This PHP-written tool allows one to store databases of a predefined format into a user-owned and password protected directory on a local SRS server [see Additional file 1] [29]. In this directory, databases can be managed, viewed and uploaded into SRS. In the "edit-database" interface, links to other databases can be specified for each field. A standard view can be generated and the location of the dataset in the SRS directory determined. Finally, permissions can be set to control access to the various datasets in SRS. Upon uploading, the Auto-Upload Tool will generate the files required for SRS: (i) the SRS data file in which the spreadsheet input file is converted into a flat-file database, (ii) an Icarus syntax file (.is-file) which describes the layout of the flat-file database, (iii) a database index file (.i-file) which describes the way in which the different fields need to be indexed for the SRS system, (iv) a database view file (.view-file) in which a standard view is defined, and (v) an information file (.it-file) which can harbour a description of the dataset. These files are automatically placed into the SRS directory after which the Auto-Upload Tool updates the srsdb.i, user.i and site.i files. These files describe the name of the database, where the files are located (srsdb.i), user permissions (user.i), and configuration of the different database groups (site.i). The srssection command within the Auto-Upload Tool implements the changes in the configuration files after which srscheck and srsdo perform indexing of new databases and set links. Incorporation of a new dataset using this tool generally takes place within minutes. On our local SRS server, a DQS (Distributed Queuing System) batch-queue is installed to prevent data loss or corruption of datasets in case multiple users are editing datasets at the same time.

### External programs accessing SRS: Venn Mapper for SRS

An important feature of storing and linking microarray data in SRS is the accessibility of the datasets for other programs. As an example, we generated a PHP web interface for the Venn Mapper program that retrieves microarray data from SRS to calculate the statistical significance of the number of co-occurring differentially expressed genes in any combination of two experiments [26]. The functionality of the original Venn Mapper was enhanced by enabling the use of different ratio cut-offs for different microarray experiments. Upon login, the interface displays all microarray databases indexed in SRS to which the user has access. After selection of the datasets a second screen shows all fields (such as individual array experiments or averaged group ratios) of the selected databases. The microarray experiments of interest can be selected for Venn Mapper analysis after which the requested data is linked and exported from SRS into the Venn Mapper program. The output of the program is available for viewing and downloading. Information requests from the interface to SRS are made through the SRS getz command [30]. This powerful feature of SRS makes the integrated databases accessible to any external program.

## Results and discussion

### Preparing and linking of microarray databases in SRS

With respect to the microarray database set-up, there are two important considerations. First, in our experience, microarray data mining often starts with selecting genes based on their differential expression. Differential gene expression is best determined using statistical evaluation of the data based on sufficient technical and biological replicates [27,28]. Dependent on the statistical test and microarray platform, raw gene expression data and/or ratio calculations can be utilized. Second, making changes to a microarray dataset in SRS is impractical and datasets should be fully built before they are imported. This means that raw expression data and ratio calculations should be normalised and flagged and represented in a common format (such as log2). Importantly, statistical evaluation should be included. For simplicity in representation, datasets can be summarised in, for example, an average of all "normal" and "cancer" samples and additional fields of log2 "normal/cancer" can be included.

**Figure 2:**
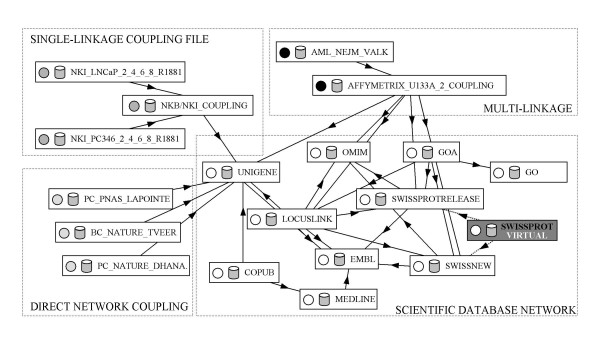
SRS Universe example view of database linkage. The scientific database network consists of well-known interlinked biological databases. Microarray datasets are coupled to this network through a multi-linked Affymetrix coupling file, the single UniGene-linked NKB/NKI-coupling file, or directly to UniGene using GenBank accession numbers. For clarity, not all databases and links mentioned are represented in the scheme. Public SRS servers and indexed scientific databases can be viewed at [43].

Linking of databases should be based on invariable and unique indexes. Links based on identifiers such as UniGene cluster identifiers that are regularly re-assigned, forces one to repeatedly update all databases that include such a denominator. Invariable links based on DNA sequence assignments such as GenBank and ProbeSet identifiers (IDs) are therefore more appropriate linking indexes. We recommend including only one of those invariable and unique linking fields in the microarray dataset to avoid the need for a regular update. Since many biological databases do not use these hard links, connecting microarray datasets to other databases can be achieved through a coupling file and/or by making use of the web of links provided by the biological databases (Figure 2). A coupling file can be minimal, containing for example only a RefSeq ID with the appropriate array spot number, or can contain a variety of indexes such as GenBank, RefSeq, SwissProt, OMIM, LocusLink, KEGG, GO, GeneCards, UniGene identifiers, that directly link to the different databases. Since some of these links are variable and biological databases keep growing with information, coupling files must be regularly renewed to update the various links.

Instead of or in combination with a coupling file, one can make use of the links provided in the various biological databases (Figure 2). For example, the GenBank accession code in the microarray dataset can be linked to the UniGene database. The LocusLink database is linked to the UniGene database through RefSeq accession numbers and also contains IDs that for example link to EMBL, OMIM, and SwissProt. In this way, almost all biological databases are linked through a network of direct and indirect connections. In case multiple roads lead to the same databases, SRS utilizes only one route. By assigning values to each link, the route taken is the one having the lowest sum of link values, even when this results in a lower number of connected fields (genes). Although SRS can be forced to take a specified path, one should be careful to be dependent on many different databases. Inconsistencies in and incompleteness of databases are accumulated when linking occurs in sequence.

Linking to the network of biological databases through a coupling file has various advantages. One can establish directly validated links to each database, including databases outside the network. In addition, errors in links can easily be corrected. Platform-specific or overall coupling files can be retrieved from the Affymetrix website [31] and from sources such as Resourcerer [32], KARMA [33], GeneHopper [34], ProbeMatchDB [35], DAVID [36], EnsMart [37], and Source [38]. Using these resources, microarray datasets from different platforms can be linked. This includes connecting cross-species datasets using ortholog converters such as HOMGL [39] and HOMOLOGENE [40].

### Gene linking efficiency of different databases

**Figure 3:**
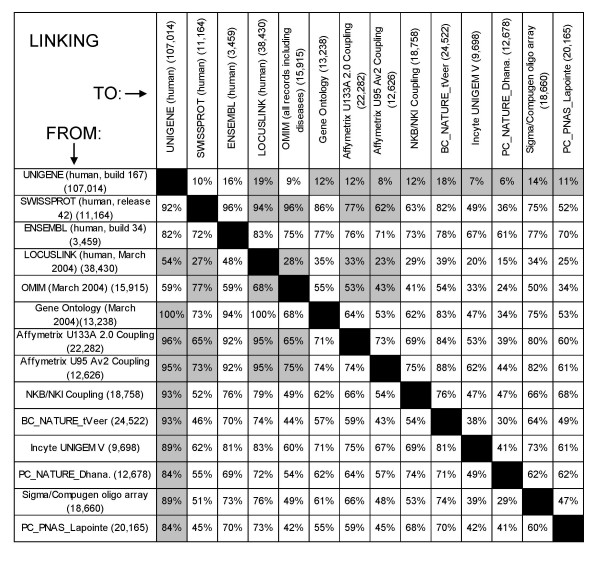
Linkage efficiency of databases within SRS. The percentage of records in a specific database (in rows) linked to others (columns) are shown. Numbers in brackets are the total number of fields in the particular database. The NKB/NKI Coupling [44], BC_NATURE_tVeer [45], Incyte Human UNIGEM V 1.0, PC_NATURE_Dhana. [46], Sigma/Compugen oligo array [47], and PC_PNAS_Lapointe [48], are linked to UniGene via a single accession code connection (see Figure 2). Direct links are depicted as grey cells. The Affymetrix U133A and U95 coupling files [31] are directly linked to different biological databases.

A high accuracy and comprehensiveness of linking are essential for a successful comparison of microarray data from different platforms. The extent of linkage of various databases in SRS was examined (Figure 3). The percentage of fields of scientific databases and microarray datasets that are linked to other databases was assessed. As shown in Figure 2, most microarray databases are directly linked to the scientific database network via a single connection. The U133A and U95 Affymetrix coupling files contain direct links to UniGene, SwissProt, LocusLink, and OMIM. The linkage of the various microarray platforms to UniGene varies between 84% and 96%. On average, 60% of the genes of microarray datasets can be linked to each other via UniGene.

### Auto-Upload Tool and external programs accessing SRS

The availability of many scientific databases in SRS, the universality of the system and its free access for academic use, make SRS an excellent mining system for heterogeneous microarray datasets. The Auto-Upload Tool facilitates the exchange of microarray datasets between separate SRS installations. Using a single data file and optional description file, any user can upload the identical data and customize it to their own SRS environment. We would urge researchers and microarray data repositories to make their data available in an SRS format. In addition, microarray software programmers could make their software available in an SRS compatible format or include SRS data export options. The commercial SRS GeneSpring^® ^Connector and public EMBOSS are examples of such microarray-SRS integration ventures.

We plan to extend our efforts of integrating more microarray databases into SRS. In addition, software tools specific for microarray data analysis, such as Go Mapper and CoPub Mapper will be rewritten for SRS [26,41,42]. The CoPub Mapper literature mining program contains databases that store, for each gene, all MEDLINE records mentioning the gene. This directly links microarray expression data to the published literature and allows for co-publication research of gene-gene and gene-keyword combinations.

## Conclusion

The Sequence Retrieval System is a versatile and useful database system to store, link and query various scientific databases, including microarray datasets. Fully processed datasets can be incorporated and linked to other datasets using the Auto-Upload Tool. This user-friendly program makes SRS accessible to users who can themselves add, link and mine databases within minutes. Datasets stored in SRS can be interrogated by external programs to perform virtually any computation.

## Availability and requirements

Project Name: Auto-Upload Tool and Venn Mapper for SRS

Project home page: 

Operating system: Platform independent

Programming language: PHP, JavaScript, Perl

Other requirements: Local SRS installation, DQS batch-queue, MySQL database server, PHP-enabled Webserver (like Apache)

License: SRS (Lion Bioscience)

Any restrictions to use by non academics: License needed

## Authors' contributions

AV and DdL generated the Auto-Upload Tool. DdL, AV and MS generated the Venn Mapper for SRS program. AV and VdJ installed and managed the servers for the various tools. Funding for the project was obtained by JK and GJ. AV, JK and GJ contributed to the intellectual content and GJ supervised the project.

## Supplementary Material

Additional File 1 describing how to use the Auto-Upload Tool program Click here for file
