# Supplementary material for: Storing, linking, and mining microarray databases using SRS
Source: BMC Bioinformatics. 2005 Jul 27;6:192. doi: 10.1186/1471-2105-6-192 (PMC1187877; doi:10.1186/1471-2105-6-192)
Supplement: Additional File 1 — describing how to use the Auto-Upload Tool program [file 1471-2105-6-192-S1.pdf]

Auto-Upload Tool Manual

Version 12 (July 20, 2005)

Antoine Veldhoven / Don de Lange / Guido Jenster
Department of Urology, Erasmus MC, Rotterdam
d.delange@erasmusmc.nl
g.jenster@erasmusmc.nl
http://www.erasmusmc.nl/gatcplatform

The Auto-Upload Tool has been published in BMC Bioinformatics (Veldhoven et al. 2005). For latest version of software and manual, please visit: **http://www.erasmusmc.nl/gatcplatform**

Contents

Introduction………………………………………………………………..... 3
Creating a data file…………………………………………………………… 4
Explanation of the data file…………………………………….. 5
Considerations………………………………………………….. 6
Using the Auto-Upload Tool…………………………………………… 7
Adding a new database………………………………………… 8
Change subsection location of database in SRS……………… 10
Create a link to other databases………………………………. 11
Setting user access……………………………………………… 12
Adding a database description…………………………………. 12
Defining a standard database view……………………………. 13
Make the dataset available in SRS……………………………… 14
Delete a dataset from SRS……………………………………… 15
Manage uploaded datasets…………………………………….. 15
Nomenclature rules Erasmus MC SRS users………………………….. 16

Introduction

The Auto-Upload Tool is a PHP-written program which allows the upload of databases into a local SRS server. SRS (Sequence Retrieval System) is a database system developed to store, link and query different types of biological databases (such as UniGene, SwissProt, MedLine, Ensembl, OMIM, LocusLink, GO, etc.). The main reason for building the Auto-Upload Tool was to allow SRS users to automatically upload microarray datasets into SRS.

Uploading datasets via the Auto-Upload Tool into SRS is a two-step process. First, the dataset is uploaded into a user-owned directory on the server, which can be viewed using “Manage Databases”. Second, the uploaded dataset needs to be adapted for its import into SRS, including steps to link the dataset, determine its section location, defining a standard view and setting user access.

The Auto-Upload Tool is locally available to Erasmus MC SRS users via the GATC Platform portal. This manual is specifically written for the Erasmus MC Auto-Upload Tool installation.
For researchers outside the Erasmus MC, the program can be downloaded for local installation (http://www.erasmusmc.nl/gatcplatform/). Setting up a local version of the Auto-Upload Tool needs to be done by an SRS administrator.

Minimal requirements include:

A local system of SRS (version 7.1.3)
A DQS batch-queue
A MySQL database server
A PHP-enabled Webserver (like Apache)

Creating a data file

The Auto-upload Tool expects a specific format of the data file in order to correctly insert the dataset into the SRS system. The data file must be a text (Tab delimited) file (.txt).

Please take a good look at the example database (Figure 1) below and take note of the Explanation of the data file, the locations and especially the “accepted characters / values”.

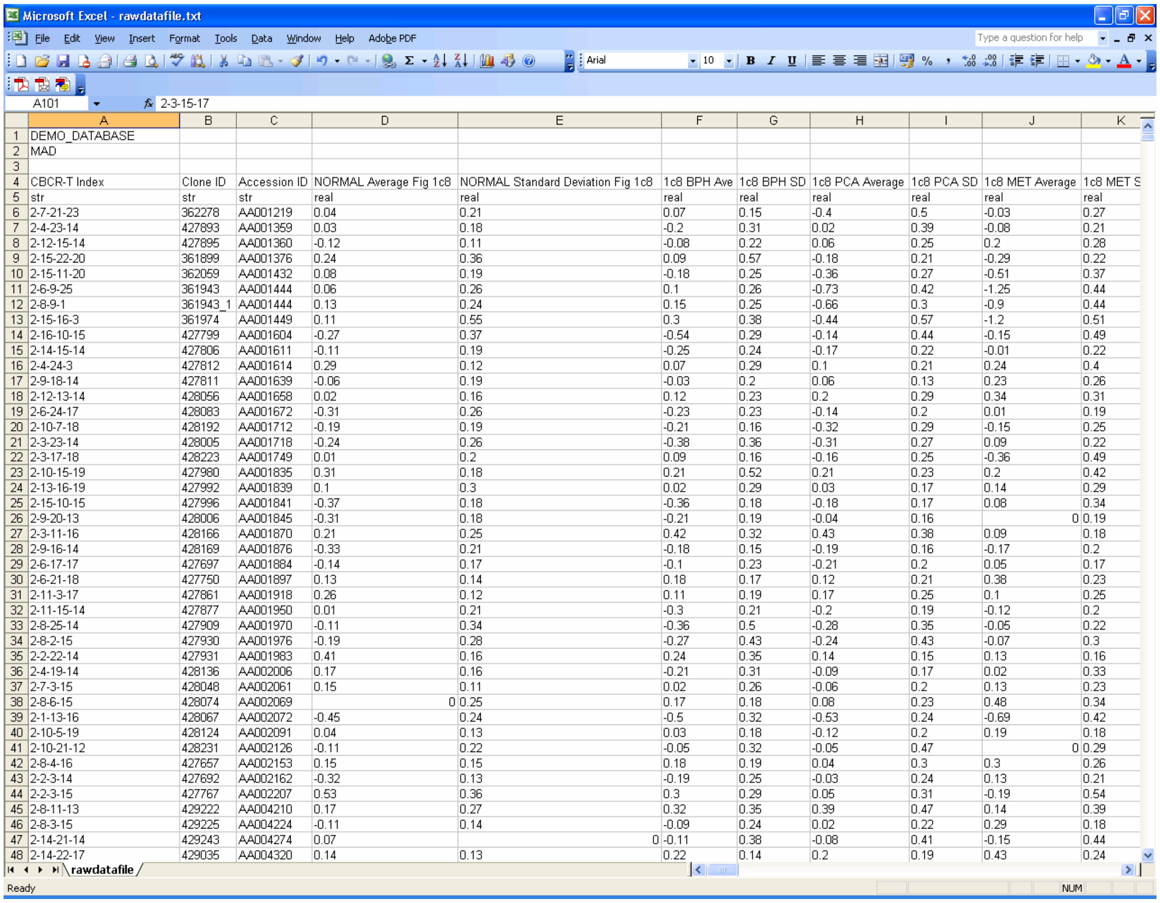

	A	B	C	D	E	F	G	H	I	J	K
1	DEMO_DATABASE										
2	MAD										
3											
4	CBCR-T Index	Clone ID	Accession ID	NORMAL Average Fig 1c8	NORMAL Standard Deviation Fig 1c8	1c8 BPH Ave	1c8 BPH SD	1c8 PCA Average	1c8 PCA SD	1c8 MET Average	1c8 MET S
5	str	str	str	real	real	real	real	real	real	real	real
6	2-7-21-23	362278	AA001219	0.04	0.21	0.07	0.15	-0.4	0.5	-0.03	0.27
7	2-4-23-14	427893	AA001359	0.03	0.18	-0.2	0.31	0.02	0.39	-0.08	0.21
8	2-12-15-14	427895	AA001360	-0.12	0.11	-0.08	0.22	0.06	0.25	0.2	0.28
9	2-15-22-20	361899	AA001376	0.24	0.36	0.09	0.57	-0.18	0.21	-0.29	0.22
10	2-15-11-20	362059	AA001432	0.08	0.19	-0.18	0.25	-0.36	0.27	-0.51	0.37
11	2-6-9-25	361943	AA001444	0.06	0.26	0.1	0.26	-0.73	0.42	-1.25	0.44
12	2-8-9-1	361943_1	AA001444	0.13	0.24	0.15	0.25	-0.66	0.3	-0.9	0.44
13	2-15-16-3	361974	AA001449	0.11	0.55	0.3	0.38	-0.44	0.57	-1.2	0.51
14	2-16-10-15	427799	AA001604	-0.27	0.37	-0.54	0.29	-0.14	0.44	-0.15	0.49
15	2-14-15-14	427806	AA001611	-0.11	0.19	-0.25	0.24	-0.17	0.22	-0.01	0.22
16	2-4-24-3	427812	AA001614	0.29	0.12	0.07	0.29	0.1	0.21	0.24	0.4
17	2-9-18-14	427811	AA001639	-0.06	0.19	-0.03	0.2	0.06	0.13	0.23	0.26
18	2-12-13-14	428056	AA001658	0.02	0.16	0.12	0.23	0.2	0.29	0.34	0.31
19	2-6-24-17	428083	AA001672	-0.31	0.26	-0.23	0.23	-0.14	0.2	0.01	0.19
20	2-10-7-18	428192	AA001712	-0.19	0.19	-0.21	0.16	-0.32	0.29	-0.15	0.25
21	2-3-23-14	428005	AA001718	-0.24	0.26	-0.38	0.36	-0.31	0.27	0.09	0.22
22	2-3-17-18	428223	AA001749	0.01	0.2	0.09	0.16	-0.16	0.25	-0.36	0.49
23	2-10-15-19	427980	AA001835	0.31	0.18	0.21	0.52	0.21	0.23	0.2	0.42
24	2-13-16-19	427992	AA001839	0.1	0.3	0.02	0.29	0.03	0.17	0.14	0.29
25	2-15-10-15	427996	AA001841	-0.37	0.18	-0.36	0.18	-0.18	0.17	0.08	0.34
26	2-9-20-13	428006	AA001845	-0.31	0.18	-0.21	0.19	-0.04	0.16	0	0.19
27	2-3-11-16	428166	AA001870	0.21	0.25	0.42	0.32	0.43	0.38	0.09	0.18
28	2-9-16-14	428169	AA001876	-0.33	0.21	-0.18	0.15	-0.19	0.16	-0.17	0.2
29	2-6-17-17	427697	AA001884	-0.14	0.17	-0.1	0.23	-0.21	0.2	0.05	0.17
30	2-6-21-18	427750	AA001897	0.13	0.14	0.18	0.17	0.12	0.21	0.38	0.23
31	2-11-3-17	427861	AA001918	0.26	0.12	0.11	0.19	0.17	0.25	0.1	0.25
32	2-11-15-14	427877	AA001950	0.01	0.21	-0.3	0.21	-0.2	0.19	-0.12	0.2
33	2-8-25-14	427909	AA001970	-0.11	0.34	-0.36	0.5	-0.28	0.35	-0.05	0.22
34	2-8-2-15	427930	AA001976	-0.19	0.28	-0.27	0.43	-0.24	0.43	-0.07	0.3
35	2-2-22-14	427931	AA001983	0.41	0.16	0.24	0.35	0.14	0.15	0.13	0.16
36	2-4-19-14	428136	AA002006	0.17	0.16	-0.21	0.31	-0.09	0.17	0.02	0.33
37	2-7-3-15	428048	AA002061	0.15	0.11	0.02	0.26	-0.06	0.2	0.13	0.23
38	2-8-6-15	428074	AA002069	0	0.25	0.17	0.18	0.08	0.23	0.48	0.34
39	2-1-13-16	428067	AA002072	-0.45	0.24	-0.5	0.32	-0.53	0.24	-0.69	0.42
40	2-10-5-19	428124	AA002091	0.04	0.13	0.03	0.18	-0.12	0.2	0.19	0.18
41	2-10-21-12	428231	AA002126	-0.11	0.22	-0.05	0.32	-0.05	0.47	0	0.29
42	2-8-4-16	427657	AA002153	0.15	0.15	0.18	0.19	0.04	0.3	0.3	0.26
43	2-2-3-14	427692	AA002162	-0.32	0.13	-0.19	0.25	-0.03	0.24	0.13	0.21
44	2-2-3-15	427767	AA002207	0.53	0.36	0.3	0.29	0.05	0.31	-0.19	0.54
45	2-8-11-13	429222	AA004210	0.17	0.27	0.32	0.35	0.39	0.47	0.14	0.39
46	2-8-3-15	429225	AA004224	-0.11	0.14	-0.09	0.24	0.02	0.22	0.29	0.18
47	2-14-21-14	429243	AA004274	0.07	0	-0.11	0.38	-0.08	0.41	-0.15	0.44
48	2-14-22-17	429035	AA004320	0.14	0.13	0.22	0.14	0.2	0.19	0.43	0.24

Figure 1: Example data file

Explanation of the data file

Please remember that in fields with descriptions (see below) only letters, numbers and the underscore (_) are allowed. **<u>Never start with a number</u>**. Do not include special characters in the description (~!@#$%^&*()-+=[]{};:”’<>?/|\, etc.).

DATABASE NAME (this will be displayed in the SRS system)

Location	**CELL A1**
Accepted characters	Numbers, Letters and ‘_’
Note	This name must be a unique name in the system.

DATABASE GROUP (this is the short name of the (sub)section in the SRS system)

Location	**CELL A2**
Accepted characters	Numbers, Letters and ‘_’
Note	May be left empty and filled in later

UNIQUE COLUMN NAME

Location	**Row # 3**
Accepted characters	Numbers, Letters and ‘_’
Note	Should be left empty, is automatically inserted

DESCRIPTIVE COLUMN NAME

Location	**Row # 4**
Accepted characters	Numbers, Letters and ‘_’

DATA TYPE

Location	**Row # 5**	
Accepted values	**int**	number (e.g. 21356 or -135636)
	**real**	floating point (e.g. -1.392 or 1.425)
	**str**	String, text values
Note	If left empty, type is set to real	

DATA

Location	**Row # 6** and onwards
Accepted values	anything goes

Considerations

- Do not include units in de data file (like %, ng/ml etc).

- Since SRS is not designed to perform calculations on the datasets, it is best to upload fully processed data, i.e. normalized, flagged and statistically evaluated ratios of microarray experiments. You can also include the raw data, like intensities, to filter for genes with an expression higher or lower than a specific intensity value.

- Gene expression ratios should be in a 2log format. Two-fold higher expression (ratio 2) will be displayed as “1”; two-fold lower expression (ratio 0.5) as “-1”; no differential expression (ratio 1) as “0”.

- Determine up-front the nomenclature of the text-variables such as database name, database group, and descriptive column names. If every researcher makes up his/her own classification, the SRS system quickly becomes a confusing mess. At the end of this manual, the “Nomenclature rules Erasmus MC SRS users” are explained.

Using the Auto-Upload Tool

Go to: http://www.erasmusmc.nl/gatcplatform/ and follow the link SRS and Auto-Upload Tool.

The following window will open:

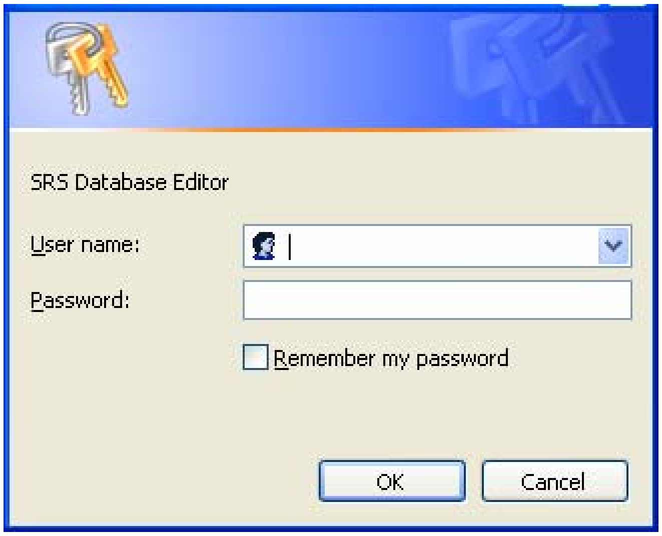

Figure 2: GATC Platform – Auto-Upload Tool login

Please fill in your SRS username & password, supplied by the SRS administrator.

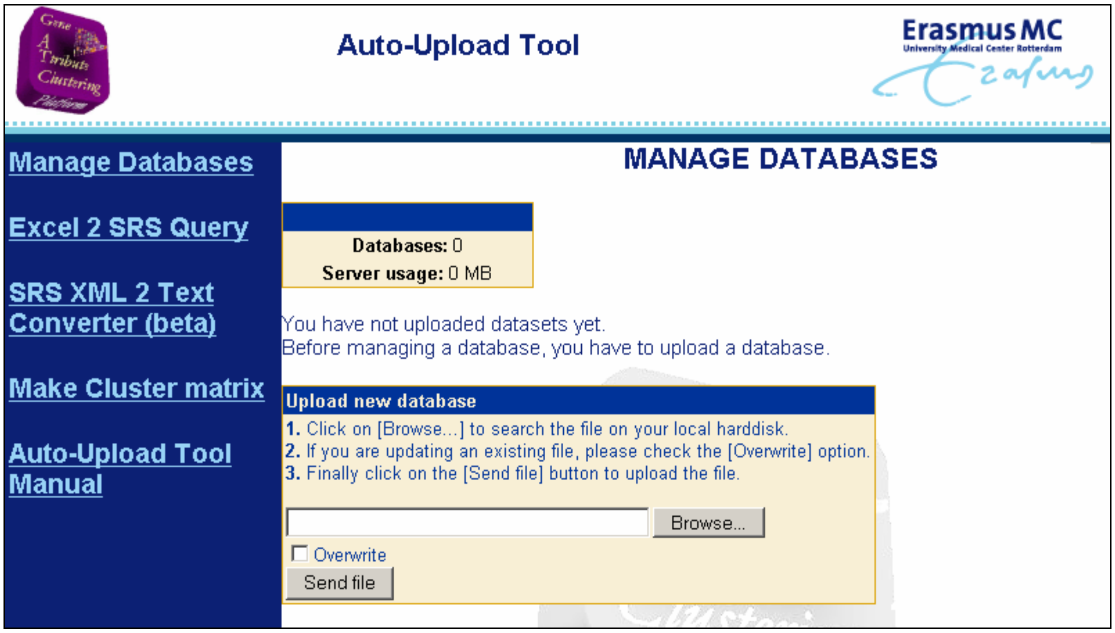

Figure 3: Initial view Auto-Upload Tool

Once logged in, the above screen appears. All your manageable databases will be listed on this page, if this is the first time you are using the system, no databases will be available.

Adding a new database

If this is the first time you are adding a database, the system will need some information from you, like an email address to communicate the results of your actions.

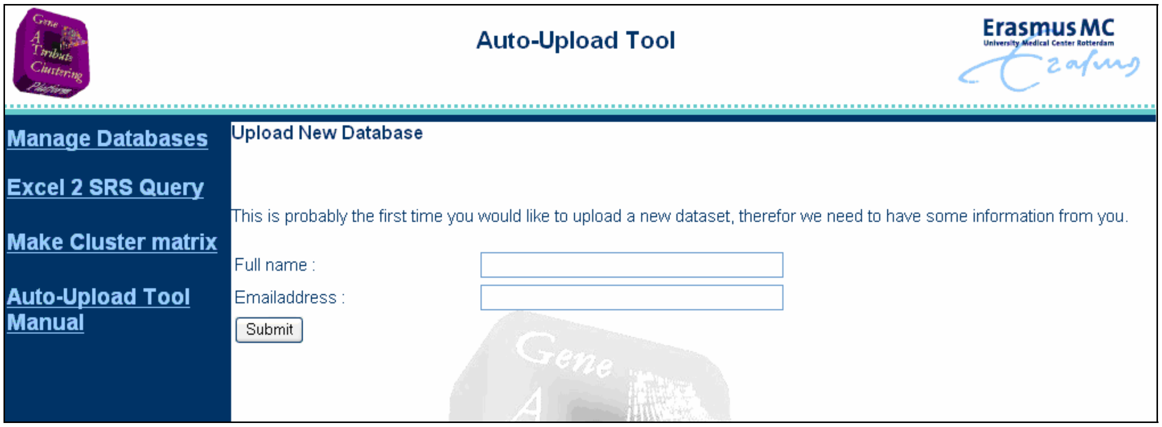

Figure 4: Providing information to the system

Please fill in your name and email address in order to receive email regarding the status of your actions.

Once your details have been filled in, you can upload your database:

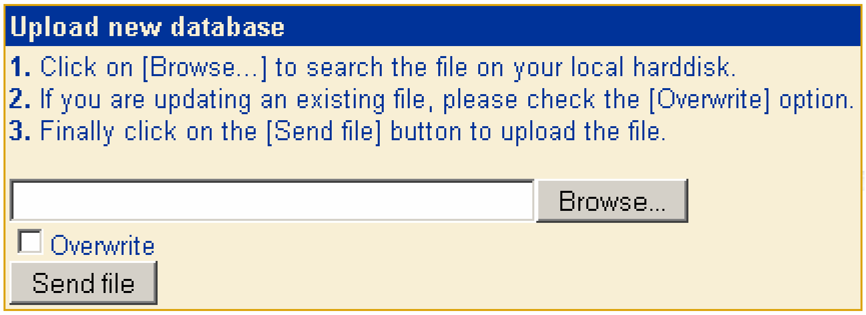

Figure 5: Upload new database

In order to upload a new database, click on browse and locate the ".txt" tab-delimited data file.

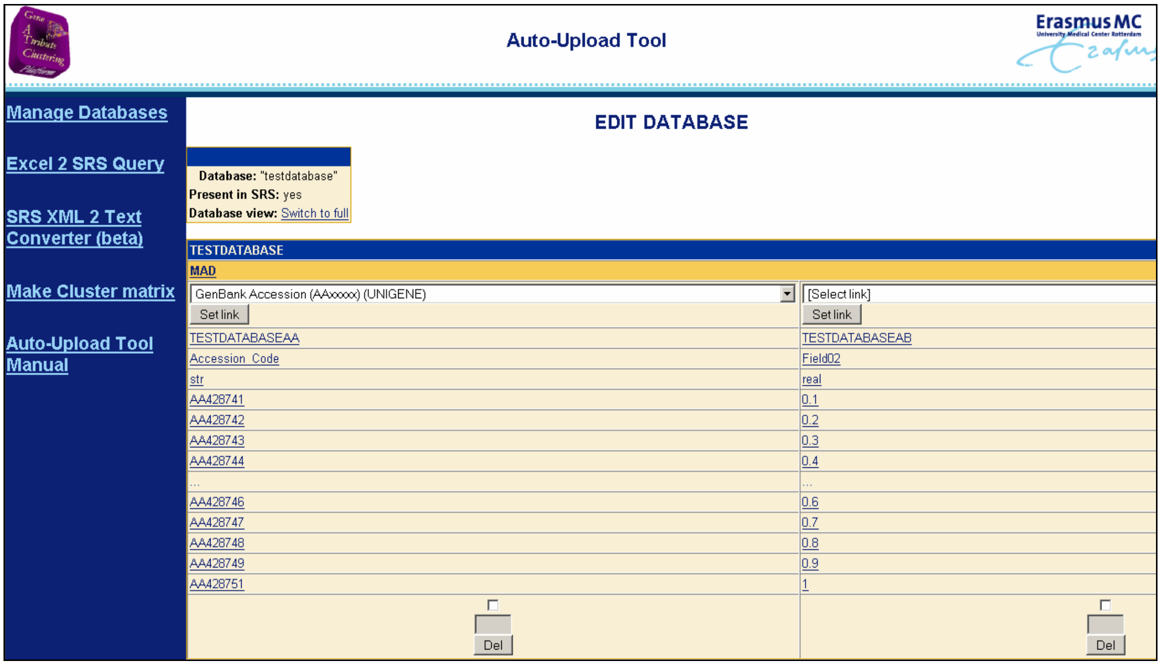

Figure 6: Edit database

This screen displays the database you have just uploaded.

You can click on any value or column name to change this value or name.

The uploaded database is placed on a user-owned section of the server. To view all uploaded databases, click on “Manage Databases”.

Change subsection location of database in SRS

Click on the entry in the second row, cell A2 (“MAD” in our example). This will open a new screen in which the subsection location of the database in SRS can be determined. Select the correct subsection and click “apply”.

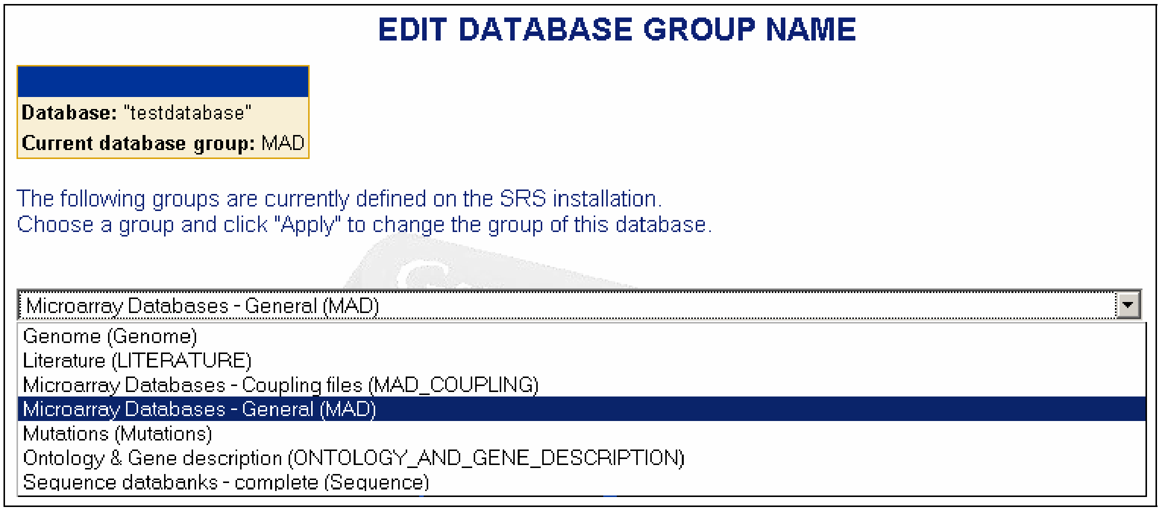

Figure 7: Selecting the location of the database in the various SRS subsections

It is essential to go through this location selection step. In case an incorrect or new subsection name is kept in cell A2, SRS will generate a new (sub)section with that particular name, disordering SRS with all kind of weird (sub)sections. In case one wants to create a new (sub)section for storing datasets, please contact the administrator first.

Create a link to other databases

Scroll to the appropriate column of your database.

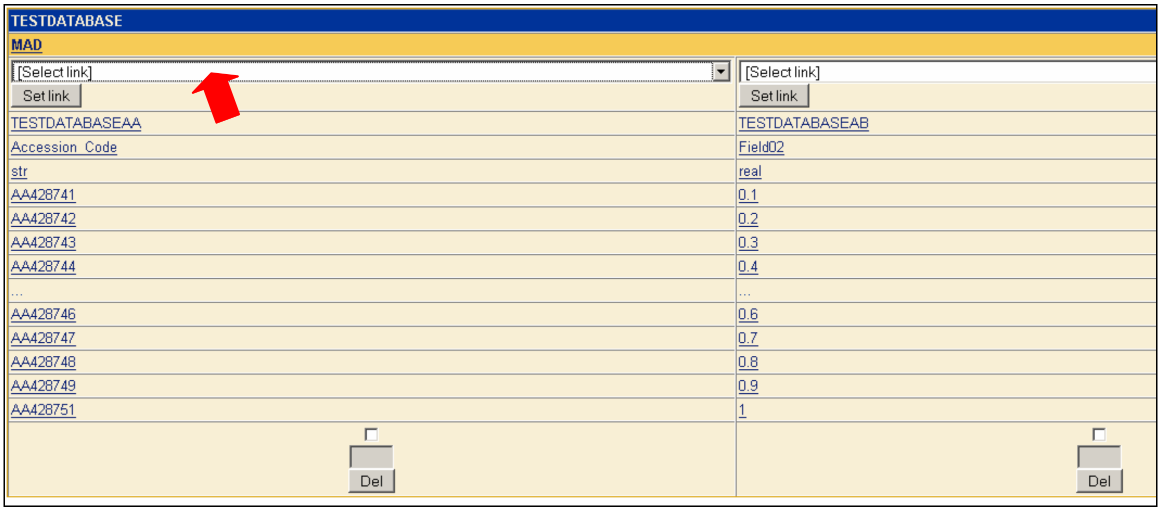

Figure 8a: Linking to UniGene through GenBank accession number

And select the appropriate linking

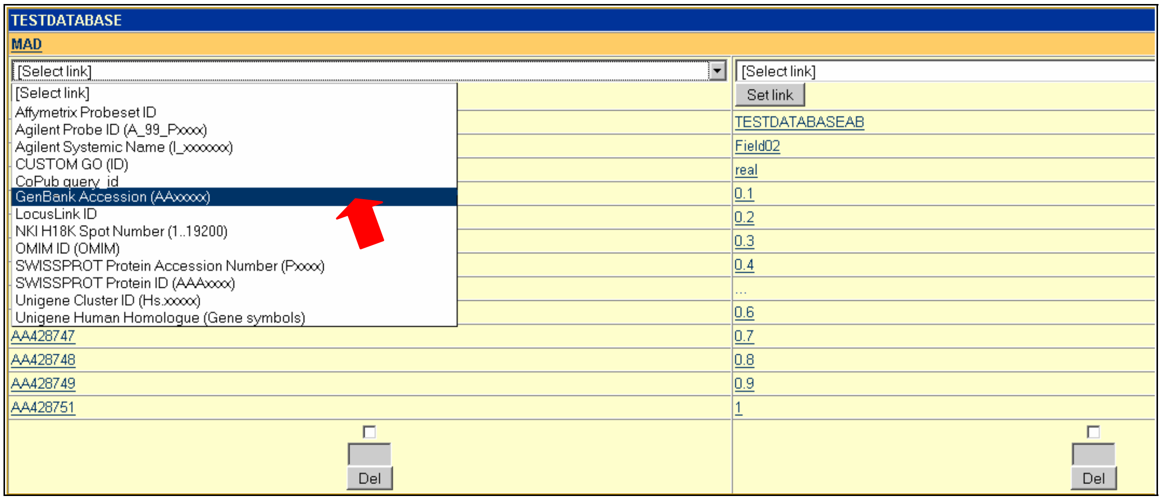

Figure 8b: Linking to UniGene through GenBank accession number

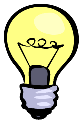

IMPORTANT:

Be sure that you press the 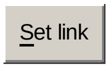

- button each time you have changed a linked column in order to save the data.

One should not link different columns (such as GenBank_Acc or UniGene_ID) to the same database (such as UniGene). Only the last indexed link will be the one used by SRS to connect your uploaded dataset with the linked database!

Setting user access

In the **Permissions** section you can decide who will have access to the database. By default this will be ONLY you.

If you UNCHECK all users, the database will become publicly available, if you don’t want to grant access to other users, please ONLY check yourself.

If the database was already uploaded into SRS, changes in permissions will automatically be implemented in SRS. It is not necessary to add the database again in SRS.

IMPORTANT:

Be sure that you press the Set permissions - button if you want to apply the permissions.

Adding a database description

The Auto-Upload Tool allows one to add a description of the dataset, which will be displayed in the SRS databank information field. The description is a text field and can be used to explain any details of the dataset. It is highly recommended to use this option to describe experimental conditions, explain abbreviations, give owner information, and more (Figure 9).
In addition to this text field, a single file can be uploaded which can be opened and viewed in the particular SRS databank information field. For example, the pdf version of the article describing the database or the grant describing the project can be attached.

Clicking the **“Save description data”** button will upload the text field and/or the attached file into the appropriate SRS databank information field. To add or adapt the text description or file, change the text or upload the new file and click the “Save description data” button. If the database was already uploaded into SRS, the description/file will be automatically replaced in SRS. It is not necessary to add the database again in SRS.

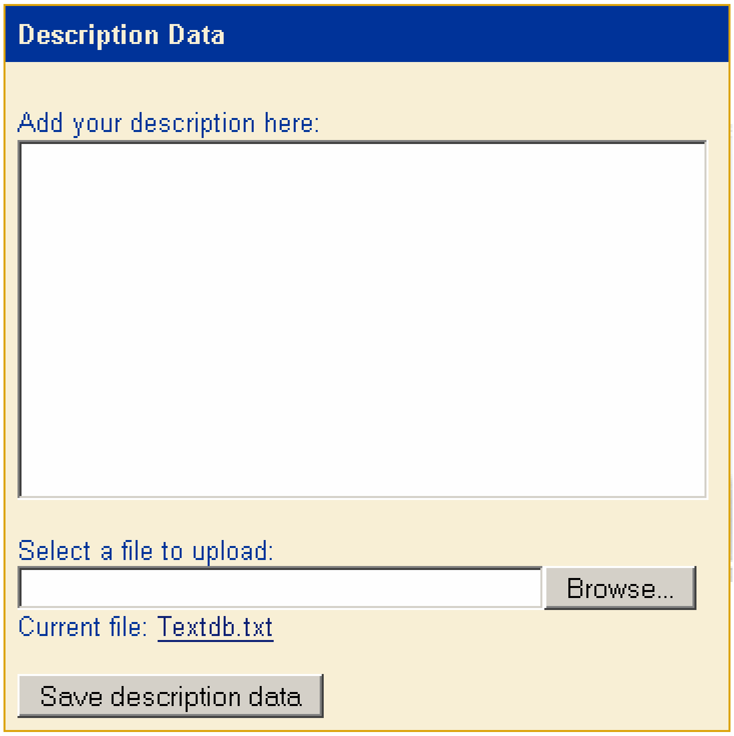

Figure 9: Adding text description and/or a single file to the database

Defining a standard database view

It is possible to define a database view in SRS which is used when you are browsing entries or viewing search results. The Auto-Upload Tool allows you to edit this view and include any database field you want, including link fields to other (common) database fields such as LOCUSLINK, SWISSPROT, OMIM and UNIGENE.

Use the checkboxes to choose the fields you want to include in your standard view.
By clicking the “Set standard view” and “Remove standard view” buttons you can add and remove the standard database view settings to and from SRS. There is no need to add the database again to SRS when changing these settings.

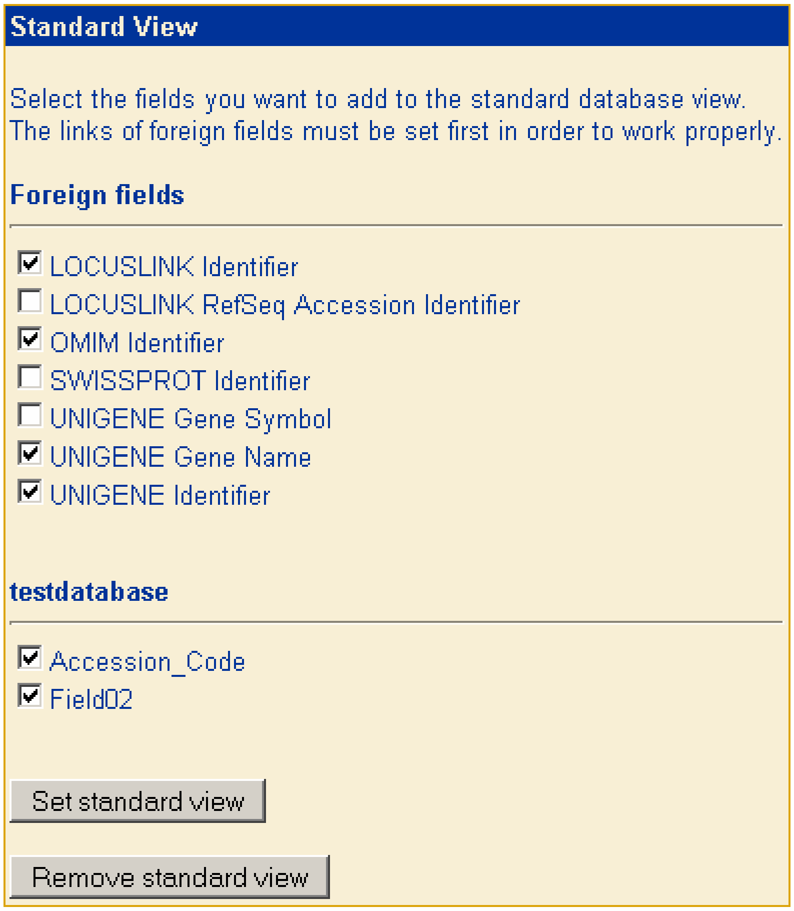

Figure 10: Defining a standard SRS view for the database

Make the dataset available in SRS

On the bottom of the page you will find the “**Add xxx to SRS**”-button.
By clicking this button, the database will be added into SRS. If the database already exists in SRS, the add-to-SRS action will overwrite the old database.

Emails will keep you informed on the performed actions and their results. Please report problems to your administrator.

Changes made to user access, the standard view, the database description, or attached file will be implemented upon clicking the appropriate buttons (“Set permissions” and “Save description data”). There is no need to again add the database to SRS (“Add xxx to SRS”). Any other changes made to the database (such as its name, SRS subsection, changing linking, changing or adding data) must be implemented by clicking the “Add xxx to SRS” button.

Delete a dataset from SRS

On the bottom of the page you will find the “**Delete xxx from SRS**”-button. By clicking this button, the database will be deleted from SRS. The database will not be deleted from the Auto-Upload Tool “Manage Database” section and can easily be uploaded into SRS again following the procedure described above.

Manage uploaded datasets

In the “Manage Databases” view, one can upload new datasets (see “Adding a new database”) and manage the uploaded datasets (Figure 11).

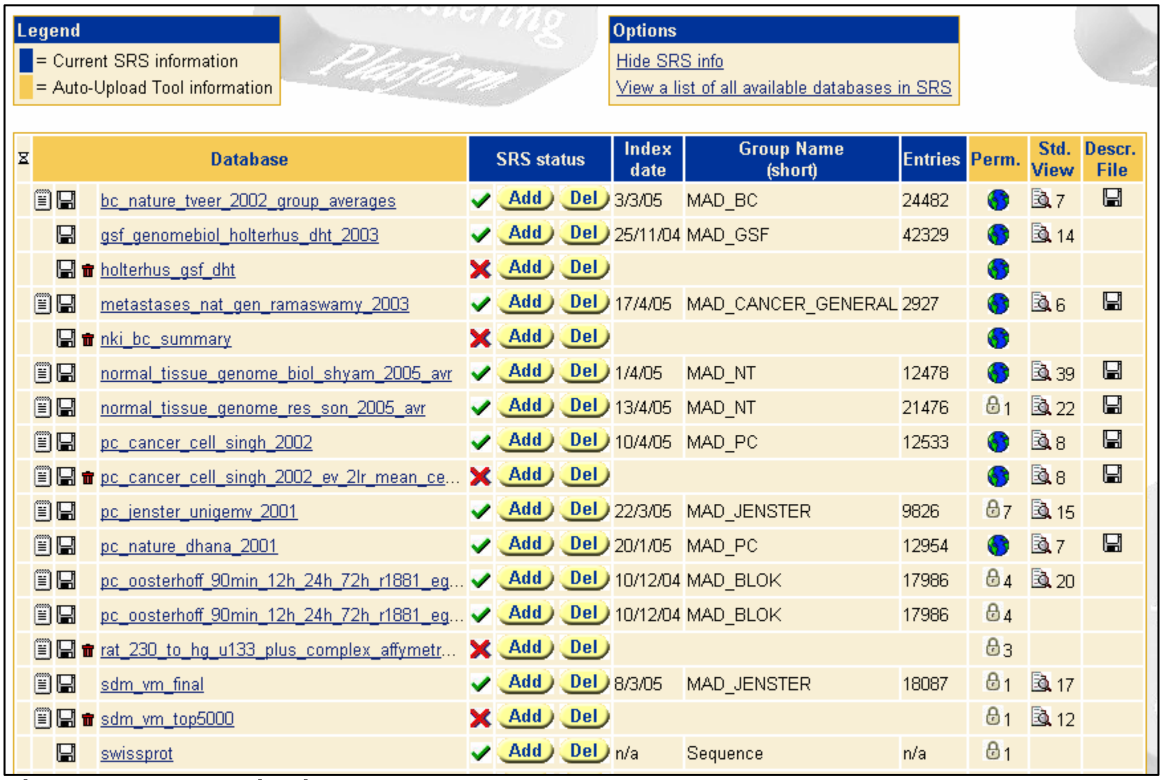

Database	SRS status		Index date	Group Name (short)	Entries	Perm.	Std. View	Descr. File
bc_nature_tveer_2002_group_averages	✓	Add Del	3/3/05	MAD_BC	24482		7	
gsf_genomebiol_holterhus_dht_2003	✓	Add Del	25/11/04	MAD_GSF	42329		14	
holterhus_gsf_dht	✗	Add Del						
metastases_nat_gen_ramaswamy_2003	✓	Add Del	17/4/05	MAD_CANCER_GENERAL	2927		6	
nki_bc_summary	✗	Add Del						
normal_tissue_genome_biol_shyam_2005_avr	✓	Add Del	1/4/05	MAD_NT	12478		39	
normal_tissue_genome_res_son_2005_avr	✓	Add Del	13/4/05	MAD_NT	21476	1	22	
pc_cancer_cell_singh_2002	✓	Add Del	10/4/05	MAD_PC	12533		8	
pc_cancer_cell_singh_2002_ev_2lr_mean_ce...	✗	Add Del					8	
pc_jenster_unigemv_2001	✓	Add Del	22/3/05	MAD_JENSTER	9826	7	15	
pc_nature_dhana_2001	✓	Add Del	20/1/05	MAD_PC	12954		7	
pc_oosterhoff_90min_12h_24h_72h_r1881_eg...	✓	Add Del	10/12/04	MAD_BLOK	17986	4	20	
pc_oosterhoff_90min_12h_24h_72h_r1881_eg...	✓	Add Del	10/12/04	MAD_BLOK	17986	4		
rat_230_to_hg_u133_plus_complex_affymetr...	✗	Add Del				3		
sdm_vm_final	✓	Add Del	8/3/05	MAD_JENSTER	18087	1	17	
sdm_vm_top5000	✗	Add Del				1	12	
swissprot	✓	Add Del	n/a	Sequence	n/a	1		

Figure 11: Manage databases

In this view, uploaded databases can be saved, viewed and changed (click on database name) and added or removed from SRS. Information on ongoing processes (such as being uploaded in SRS), number of entries, group name, permissions, standard view and description file are displayed.

Nomenclature rules Erasmus MC SRS users

Database name (cell A1)

1) published microarray databases
The database name for published microarray datasets should reference the disease or tissue, the journal, first author, and year of publication.
Examples:
PC_PNAS_LAPOINTE_2004
BC_NATURE_TVEER_2002
AML_NEJM_VALK_2004

2) unpublished microarray databases
The database name for user-owned unpublished datasets should reference the disease or tissue and conditions and can include researcher's name, microarray platform and date/year.
Examples:
NKI_H18K_LNCaP_2_4_6_8_R1881
PROSTATE_ZONES_18K_oligo_2003

3) Coupling files
Coupling files will generally be constructed and updated by the SRS administrator and have the format: microarray platform, subtype and "coupling".
Examples:
AFFYMETRIX_U133A_2_COUPLING
NKB/NKI_HUMAN_18K_cDNA_COUPLING

Database group (cell A2)

Any public microarray database should be deposited under the section:
"Microarray Databases – General – "subsection"

User-owned microarray datasets should be deposited under the user's name:
"User owned Databases – *User Name*"

Coupling files are uploaded in the "Microarray Databases – Coupling Files" section.

Descriptive column names (row #4)

Column names, also referred to as "field names" include the standard field names and experiment names.

Standard column names should be kept to a specific nomenclature:

Description	Mandatory column name
Unique SRS identifier	ID (automatically generated by SRS)
GenBank accession number	GenBank_Acc
UniGene cluster ID	UniGene_Hs (or UniGene_Mm, etc.)
Full gene name	Gene_name
Gene symbol	Gene_symbol
Omim ID	OMIM
LocusLink ID	LocusLink
RefSeq ID	RefSeq
SwissProt ID	SwissProt
Affymetrix Probeset ID	Affy_U95_Av2_ID or Affy_U133A2_ID, etc.

Experiment column names should be as descriptive of the experiment as possible and they MUST indicate the type of data. Some examples:
LNCaP_2h_R1881_2LR_AVR
PC82_Cas_SD_perc
Avoid non-descriptive phrases like: TEST_1; Sample_34; P13_2

The different abbreviations representing the different types of data:

- expression values……………………………..	_EV
- normalized……………………………..	_EV
- raw data………………………………..	_Raw_EV
- Cy3 or Cy5…………………………….	_CY3/5_EV
- Affymetrix………………………………	_Aff_EV
- background values or other…………	_bckg_EV
- averages	_EV_AVR
- averages of all arrays	_EV_AVR_ALL
- 2log ratios…………………….………………..	_2LR_
- averages of arrays (e.g. dye swaps)…	_2LR_AVR
- averages of larger groups…………….	_2LR_GR_AVR
- averages of all	_2LR_AVR_ALL
- mean or median centered…………….	_2LR"_xx"_centered
- standard deviation or error……………………	_SD
- as percentage…………………………	_SD_perc
- counts (e.g. # times flagged)………………..	_count
- as percentage	_count_perc
- SAM or other statistical values……………….	_SAM
- yes or no statistically significant………	_SAM_count
